# Cutaneous Manifestations of Liver Disease: A Narrative Review

**DOI:** 10.7759/cureus.70357

**Published:** 2024-09-27

**Authors:** Sofía Martínez Jiménez

**Affiliations:** 1 Internal Medicine, Centro Médico Nacional Siglo XXI, Mexico City, MEX

**Keywords:** dermatology, liver diseases, metabolic, skin diseases, skin manifestations

## Abstract

Chronic liver disease is a major cause of morbidity and mortality. The most common extrahepatic manifestations are dermatological. The pathophysiology of these dermatological manifestations is not clear, but it is postulated that the mechanisms involved include generalized vasodilatation, hyperdynamic blood circulation, and altered estrogen metabolism. The most common cutaneous manifestations of liver disease are Terry’s nails and pruritus. Terry’s nails consist of leukonychia with a distal pink band and the absence of the lunula. The main differential diagnosis is Lindsay’s nails. Vascular manifestations of liver disease include palmar erythema and arachnoid nevi, the latter located in the vascular territory of the superior vena cava and occurring in chronic alcohol-associated liver disease. Dermatological manifestations generally resolve with improvement or remission of liver disease, and there is no specific treatment for them. However, bile acid chelators are the first line of treatment for cholestatic pruritus. Studying dermatological manifestations of liver disease contributes to early diagnosis and treatment, potentially improving the patient’s prognosis.

## Introduction and background

Chronic liver disease is a significant contributor to global morbidity and mortality [1]. The primary cause of mortality in patients with liver disease is cirrhosis, with chronic hepatitis B virus infection, hepatitis C virus infection, alcoholic liver disease, and non-alcoholic steatohepatitis being the most common etiological factors [2].

The most severe morbidity and mortality associated with cirrhosis are observed in the decompensated subtype, which is characterized by ascites, esophageal variceal bleeding, encephalopathy, and elevated bilirubin levels. The annual rate of transition from compensated to decompensated cirrhosis ranges from 4-10%, leading to a notable increase in mortality rates [3].

The identification of compensated cirrhosis is crucial, as delayed intervention increases the risk of progression to the decompensated stage. Although cirrhosis is diagnosed histologically, clinical assessment, laboratory tests, and imaging studies play vital roles in daily practice.

Dermatological manifestations are often the primary or most prominent extrahepatic signs of liver disease [4]. Most of these manifestations are asymptomatic and do not require specific treatment. However, recognizing them aids in the diagnosis and management of the underlying liver condition [5]. The cutaneous manifestations of liver disease result partly from generalized vasodilation and disruptions in estrogen metabolism, leading to elevated serum estrogen levels in patients with liver impairment [6,7].

The following are some of the dermatological manifestations of liver disease: pruritus, dermatologic vascular manifestations associated with liver conditions (palmar erythema (liver palms), spider angiomas, head of Medusa), jaundice, hyperpigmentation, coagulation abnormalities associated with liver conditions (petechiae, ecchymoses, mucosal bleeding) and nail changes associated with liver conditions (Terry's nails, Muehrcke's Lines, clubbing, onycholysis).

Given the high incidence and substantial morbidity and mortality associated with these manifestations, this narrative review aimed to explore the clinical features and frequency of dermatological signs in cirrhosis and liver disease to enhance medical practice by facilitating timely and comprehensive management.

## Review

Methodology 

A literature search was conducted in the PubMed, ClinicalKey, and Science Direct databases utilizing the following search strategy: ("Skin"[Mesh] OR "Skin Manifestations"[Mesh] OR "Skin Diseases"[Mesh]) AND "Liver Diseases"[Mesh]. Publications written in English and Spanish and published up to March 2024 were reviewed. Additionally, publications sourced through manual search from references cited in review articles were included. A total of 27 publications were consulted; the information was synthesized and presented via a narrative review.

Results

Pruritis

It is the most common cutaneous manifestation of chronic liver disease. It initiates and predominates in palms and soles, potentially extending to the back, abdomen, and anterior regions of the legs [8]. Its course is marked by intermittent and chronic patterns, exhibiting a circadian rhythm, with exacerbations in the evening [9]. Scratching of the skin precipitates secondary lesions such as excoriation and lichenification.

Its prominence is observed in obstructive and cholestatic biliary pathologies, including primary biliary cirrhosis (present in 70% of the patients), sclerosing cholangitis, biliary obstruction secondary to calculi, and bile duct carcinoma. The foremost factors linked to pruritus in liver disease encompass active hepatitis B virus infection, diabetes, and elevated aspartate aminotransferase (AST) levels exceeding 60 U/L [5-7,9,10].

The prevailing theories concerning pruritus in the context of chronic liver disease are as follows.

Bile salt accumulation triggers mast cell degranulation, while bile acids induce hepatocyte injury leading to the release of pruritogenic substances [9].

Autotaxin, an enzyme responsible for lysophosphatidic acid synthesis, is associated with pruritus intensity [9].

According to the clinical practice guidelines outlined by the European Association for the Study of the Liver (EASL), primary therapy entails bile acid sequestrants such as cholestyramine administered at doses ranging from 4 to 16 grams daily. Tminimize interference with intestinal drug absorption, this medication should be administered at least four hours apart from other medications [11]. Principal limitations include adverse effects such as constipation and fat malabsorption [9-12]. Rifampicin, ranging from 150 to 300 mg daily, is advocated as a second-line therapy by the EASL. By inducing enzymatic activity (CYP3A4), rifampicin influences the metabolism and elimination of potential pruritogens while concurrently reducing autotaxin expression. Notably, its use is associated with hepatotoxicity, necessitating the monitoring of liver function tests 6-12 weeks post-initiation or dose alteration. Moreover, rifampicin administration perturbs vitamin K metabolism, leading to an increase in the international normalized ratio [9-12].

Oral opioids such as naltrexone and nalmefene constitute third-line therapeutic options. Naltrexone is typically initiated at doses ranging from 25 to 50 mg daily, with gradual titration to mitigate potential opioid withdrawal-like reactions during the initial treatment phase [11,12].

Antihistamines have demonstrated limited efficacy in alleviating hepatic pruritus. Although first-generation antihistamines may ameliorate nocturnal pruritus owing to their sedative properties, their use is discouraged in liver diseases, particularly autoimmune liver conditions, where sedation may exacerbate existing fatigue [12].

Dermatologic Vascular Manifestations Associated With Liver Conditions

Dermatological manifestations secondary to vascular alterations include palmar erythema, spider nevi (telangiectasias), and a mottled appearance resembling “paper money” skin.

**Figure 1:**
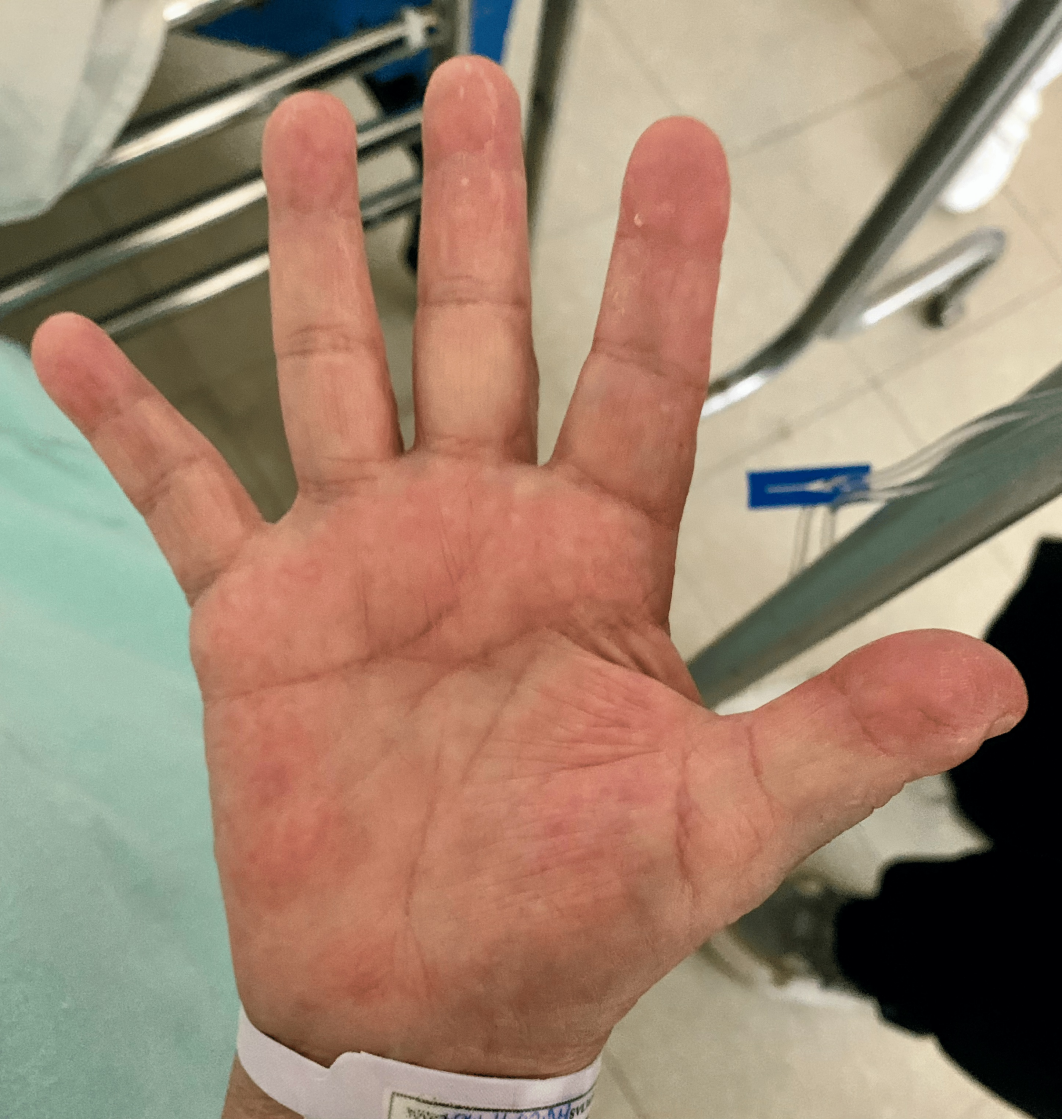
Palmar erythema in a female patient hospitalized with hepatocellular carcinoma, secondary to grade III ascites, diagnosed six months prior. Palmar erythema is observed primarily on the thenar and hypothenar eminences, as well as on the fingertips. This differs from physiological erythema, which is distributed across the entire palm and is associated with specific positions, high temperature, or pressure [5]. Image credits: M.D. Sofía Martínez

Palmar Erythema (Liver Palms): It manifests as a symmetrical, blanchable, painless erythema, primarily affecting the hypothenar eminence, thenar eminence, and fingertips (Figure 1).

It is the result of increased serum estrogen, vascular endothelial growth factor (VEGF), and substance P. Abnormal serum levels of estradiol activate the nitric oxide synthase enzyme to produce nitric oxide and induce vasodilation. Liver disease presents with a prevalence of 23%, affecting two-thirds of patients with cirrhosis. Notably, among individuals with liver disease, palmar erythema exhibits a positive likelihood ratio of three point seven [4-7].

Spider Angiomas: Spider angiomas consist of a central papule (0.5 to 1 mm) corresponding to a dilated arteriole, from which capillaries radiate peripherally. Compression of the central vessel causes temporary blanching and disappearance of the lesion, while decompression leads to rapid refilling of the peripheral vessels from the central arteriole. The central arteriole resembles a spider’s body, and the radiating vessels resemble spider legs. These lesions are typically located in the territory of the superior vena cava, primarily on the neck and face [3-5].

They are present in 10-15% of healthy individuals, especially in children and women using estrogen. In liver disease, their prevalence is 33% [5]. The number and size of spider nevi correlate with the severity of liver disease, with increased estrogen and vasodilation contributing to their development. They are particularly characteristic of alcoholic cirrhosis, likely due to the effects of VEGF and ethanol-induced angiogenesis. These lesions generally regress with the resolution of the underlying liver disease or after liver transplantation [7,13].

“Paper money” skin is a rare variant of spider angiomas characterized by thin, diffuse plaques of superficial capillaries located in the territory of the superior vena cava [5]. The thin vessels resemble the silk threads found on U.S. dollar bills.

Head of Medusa: Severe portal hypertension can induce collateral circulation, resulting in esophageal, gastric, abdominal, and rectal varices. In the context of portal hypertension, the umbilical vein, which is typically obliterated in early childhood, may recanalize. Blood from the portal venous system is diverted through the periumbilical veins to the umbilical vein and subsequently to the veins of the abdominal wall, making them prominent [5,14].

**Figure 2:**
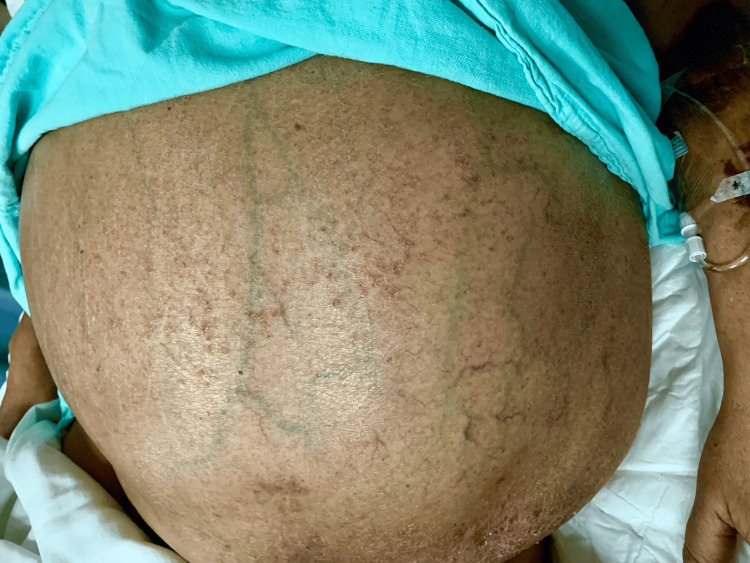
Head of Medusa observed in a male patient diagnosed with hepatocellular carcinoma, hospitalized for bleeding esophageal varices one year after the oncological diagnosis. The direction of venous flow provides valuable diagnostic information regarding venous etiology. The head of Medusa sign is most commonly associated with portal hypertension. In cases of portal hypertension, the venous flow extends both caudally and cranially from the umbilicus. In individuals with liver disease, the likelihood ratio for cirrhosis of 9.5 [3]. Image credits: M.D. Sofía Martínez.

The sign of the head (caput) of medusa refers to the abdominal varicose veins radiating from the navel to the periphery through the abdominal wall (Figure 2).

**Figure 3:**
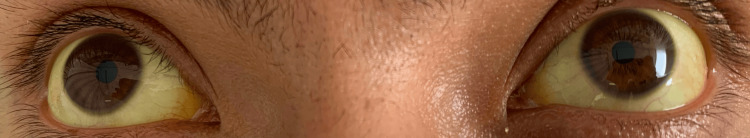
Scleral icterus observed in a 30-year-old male patient hospitalized owing to jaundice. Uniform scleral icterus was observed. The patient depicted in the figure was hospitalized for jaundice syndrome. Serological tests conducted during hospitalization identified a chronic hepatitis B virus infection as the underlying cause. Image credits: M.D. Sofía Martínez.

Jaundice: Jaundice is the yellow pigmentation of the skin, sclera, and mucous membranes resulting from the accumulation of bilirubin and its metabolites in the tissues. It manifests uniformly and becomes evident when the total serum bilirubin concentration exceeds 2 mg/dl (34 mmol/l). In liver disease, it is associated with an odds ratio for cirrhosis of 3.8 (Figure 3) [3].

Pigmentation: In patients with cirrhosis, irregular or patchy grey pigmentation may occur, either confined to the mucous membranes or diffusely distributed, particularly in cases of long-standing cirrhosis [15].

Additionally, hyperpigmentation in the anterior tibial region is observed owing to erythrocyte extravasation and hemosiderin deposition resulting from limb edema and venous flow obstruction [5].

Coagulation Abnormalities Associated With Liver Conditions

Thrombocytopenia: Thrombocytopenia, defined as a platelet count below 150,000/µL, is the most common hematological abnormality in patients with chronic liver disease. Hypersplenism plays a crucial role in thrombocytopenia induced by chronic liver diseases. Portal hypertension leads to the redistribution of splanchnic venous flow, resulting in congestion and enlargement of the spleen, which leads to splenic sequestration of platelets [16].

**Figure 4:**
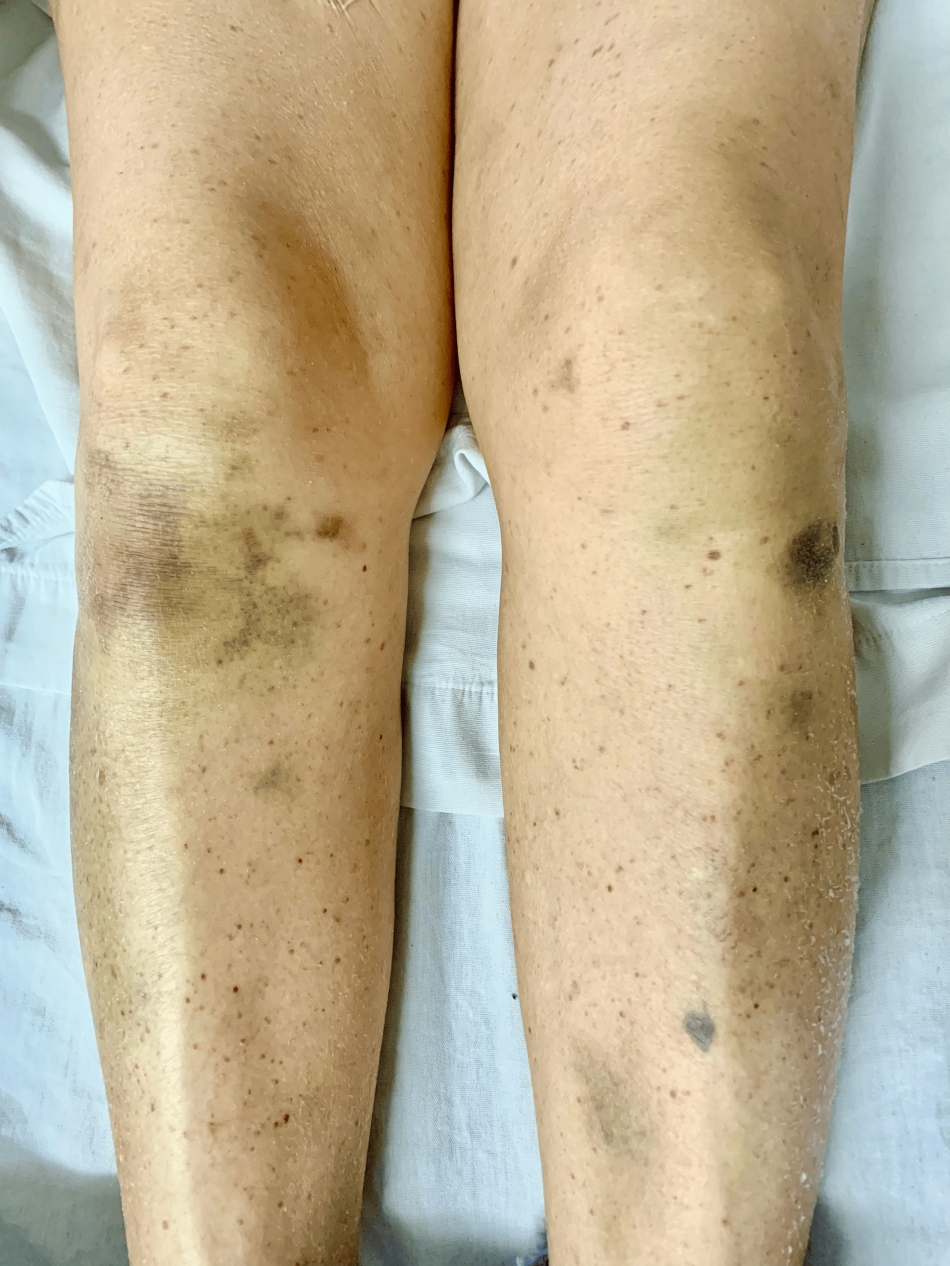
Petechiae and ecchymoses observed in a 68-year-old female patient diagnosed with chronic liver disease secondary to metabolic dysfunction-associated fatty liver disease hospitalized owing to variceal bleeding. Image credits: M.D. Sofía Martínez.

Petechiae, ecchymoses, and mucosal bleeding are indicative of coagulation disorders associated with cirrhosis (Figure 4) [5].

Nail Changes Associated With Liver Conditions

Terry’s Nails: Terry's nails exhibit a prominent leukonychia covering >80% of the nail surface, indicating connective tissue hyperplasia [17,18].

**Figure 5:**
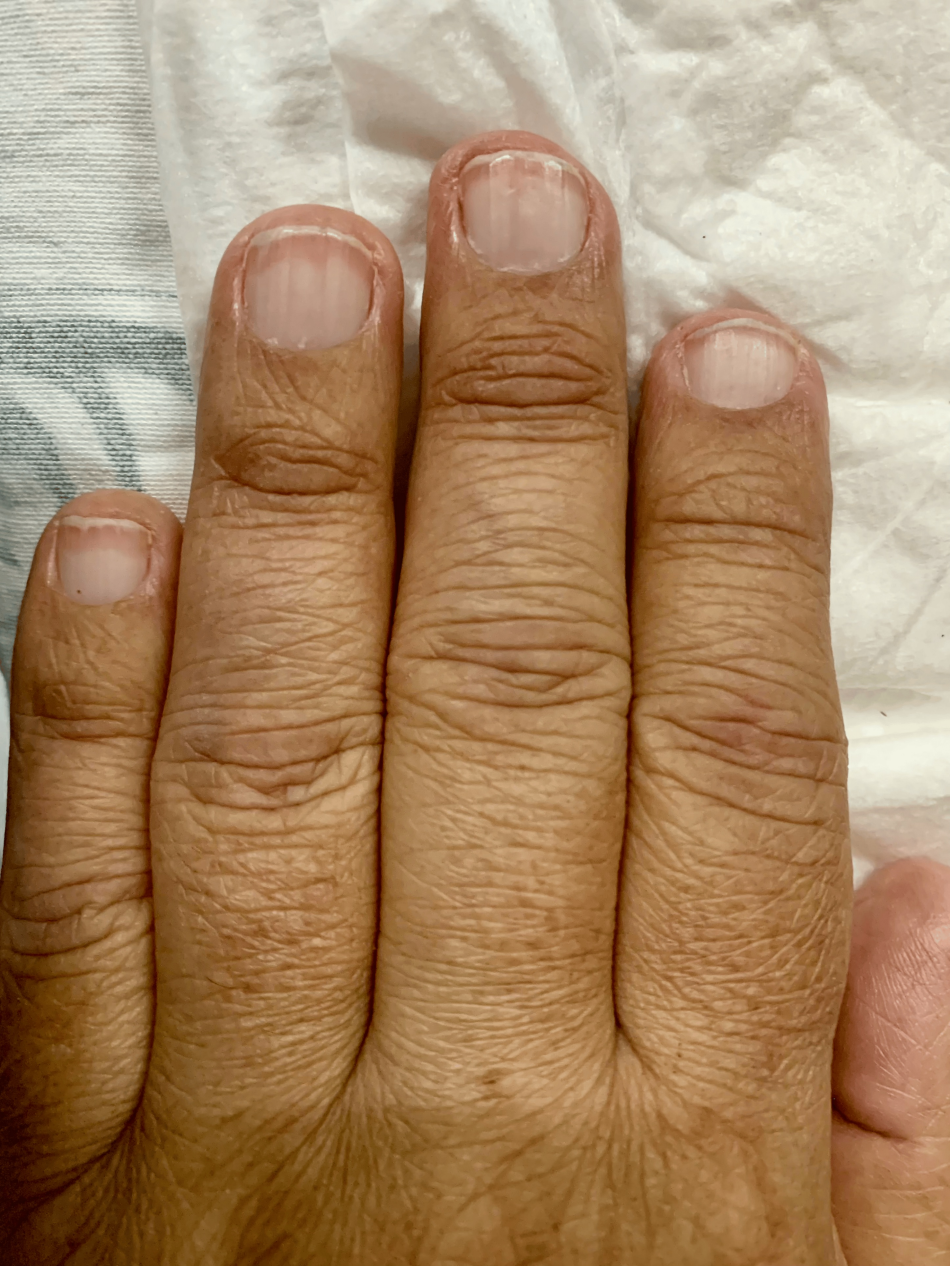
Terry’s nails observed in a male patient diagnosed with hepatocellular carcinoma hospitalized owing to bleeding esophageal varices, one year post-oncological diagnosis. Terry’s nail appear as a noticeable proximal leukonychia. They are predominantly observed on the first and second nails of the hands. Leukonychia accompanied by a distal pink band can be observed in the image. Image credits: M.D. Sofía Martínez.

These nails feature a distinct distal band of pink or brown hue, measuring 0.5 to 3 mm in width, suggesting vascular dilation and telangiectasias. Furthermore, the lunula is absent. They present with a characteristic ground-glass appearance secondary to increased connective tissue in the nail bed (Figure 5) [5,19,20].

Their pathophysiology is not well understood; however, altered metabolism may induce vasodilation and overgrowth of characteristic connective tissues [18].

The main differential diagnosis is Lindsay’s nail, characterized by the distal extension of the lunula covering more than 50% of the nail bed. They result from increased concentrations of melanocyte-stimulating hormones and are observed in chronic kidney disease [5,21].

They are observed in 40% of patients with liver disease. Soomro et al. reported that Terry’s nail is the most common dermatological finding in liver disease [18,22]. Sack et al. documented that hepatic cirrhosis was significantly correlated with Terry’s nails, irrespective of the etiology of liver disease, exhibiting an odds ratio of 5.7 (P < 0.0001). The sensitivity and specificity of Terry’s nail in detecting cirrhosis were 25.8% and 92.7%, respectively [23].

Muehrcke’s Lines: These are narrow, arched, white transverse bands that run parallel to the lunula. They disappear when pressure is applied and represent apparent leukonychia. They are most prominent on the second, third, and fourth handnails [5,20].

They are characteristic of nephrotic syndrome and manifest as states of malnutrition and liver disease. They are also associated with hypoalbuminemia (albumin concentration less than 2.2 g/dL). However, its pathophysiology remains unclear. It is hypothesized that edema in the nail bed increases the pressure on the vasculature in this region. Consequently, a normal erythematous appearance is not observed through the nail plate [20]. Nonetheless, they resolve with the normalization of albumin levels [24].

**Figure 6:**
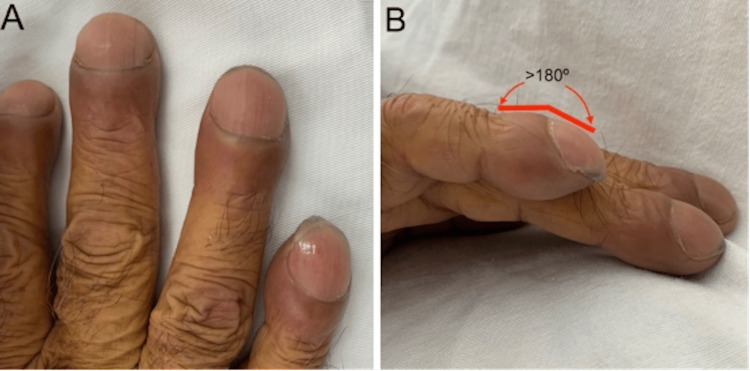
Clubbing observed in a 68-year-old male patient diagnosed with pancreatic head cancer hospitalized owing to jaundice syndrome lasting for six months. In Figure 6A, “watch glass” nails can be seen. In Figure 6B, Lovibond’s angle is depicted in a patient with acropathy. The Lovibond angle is formed by the proximal skin of the cuticle and nail bed. It is considered pathological and characteristic of acropachy when it exceeds 180º. The anatomical Lovibond angle is typically less than 165 degrees [5]. Image credits: M.D. Sofía Martínez.

Clubbing: These are convex nails (watch-glass nails), resulting from an increase in both the transverse and longitudinal curvatures of the nail. There are clinical criteria for differentiating between clubbing and pseudo-clubbing. Notably, in clubbing, the Lovibond angle is greater than 180° (Figure 6) [5].

True acropachy manifests as a positive Schamroth sign, characterized by obliteration of the typical rhomboidal space formed when aligning the symmetrical dorsal surfaces of the terminal phalanges. The skin covering the base of the nail is thin and shiny. Approximately 15% of patients with liver disease develop acropachy. Acropachy has been observed in neoplastic, pulmonary, digestive, and cardiac pathologies. It results of dilated digital arteriovenous anastomosis and increased blood flow. The pathogenesis remains unclear; however, the currently accepted hypothesis suggests that the abnormal expression of fibroblast growth factors ultimately induces the production of VEGF and platelet-derived growth factor. These factors collectively contribute to the typical findings of acropathy, such as edema, vascular hyperplasia, fibroblast proliferation, and collagen synthesis. Additionally, VEGF serves as a potent osteogenic factor [4,25,26]. 

Onycholysis: Onycholysis refers to the detachment of the nail bed from the nail plate. Typically originating at the distal edge, it has a whitish appearance owing to the air trapped beneath the separation [27].

This condition has various causes, including hyperhidrosis, infections, systemic diseases, and medications (tetracyclines, capecitabine, five-fluorouracil, olanzapine, griseofulvin, and oral contraceptives). The involvement of multiple nails suggests a systemic etiology[A6] . In adults, hyperthyroidism is the most common systemic cause of onycholysis; other systemic causes include psoriasis, scleroderma, sarcoidosis, and systemic lupus erythematosus. Additionally, there was a significant association between liver cirrhosis and onycholysis [5,27].

Hair

Alterations result from an imbalance between estrogen and testosterone levels [27]. In men, cirrhosis causes decreased facial hair growth, gynecoid pubic hair patterns, testicular atrophy, oligospermia, and gynecomastia [5].

Discussion

Most dermatological manifestations of liver disease do not correspond to specific liver disease manifestations but are present in various pathologies. However, dermatological manifestations associated with liver disease can be the first indications of liver disease in a patient. Moreover, certain dermatological manifestations may provide valuable clues regarding the etiology or severity of liver disease. Consequently, monitoring these manifestations can be beneficial for healthcare professionals in assessing treatment responses. It is noteworthy that certain manifestations commonly observed in Caucasian populations, such as spider angiomas and the characteristic blue lunula of Wilson’s disease, are less prevalent in populations with darker skin owing to inherent skin characteristics and geographical factors.

In recent years, advancements in primary preventive measures and treatments for hepatitis B and C, along with an increase in obesity and cardiovascular diseases, have led to an increase in the prevalence of non-alcoholic steatohepatitis (NASH). Since 2017, NASH has become the leading cause of cirrhosis [2]. Recent studies on the global burden of disease indicated higher mortality rates related to NASH in Latin America [2]. Hence, there is a call for future research to evaluate risk factors and associated conditions to better understand and address the reported prevalence and morbidity in our population.

Detecting liver disease in its early stages benefits the patient's quality of life and prognosis. Additionally, timely detection would represent a reduction in the burden on healthcare services worldwide. Therefore, timely diagnosis and treatment would benefit both patients and the public health system.

Although statistical association measures were employed to assess the diagnostic value of the described physical findings for cirrhosis or liver disease, knowledge regarding the correlation between dermatological manifestations and liver disease remains limited. Owing to the high prevalence and prognosis of liver disease, there is a clear need for additional research on a global scale.

## Conclusions

Chronic liver disease is a multifaceted clinical condition. In the initial compensated stage, it is typically asymptomatic. Understanding the dermatological manifestations of liver diseases, which are the most common extraintestinal presentations, facilitates early diagnosis and treatment, thereby reducing the likelihood of associated complications. The clinical characteristics of these manifestations are well-documented in the medical literature, and a meticulous examination of these features would enhance decision-making in routine clinical practice and can lead to better patient outcomes. Similarly, a meticulous physical examination helps clinicians conduct an exhaustive differential diagnosis.

Given the impact of chronic liver disease on the productive-age population and its high morbidity and mortality rates, it is considered a significant public health concern. To formulate effective and resource-efficient policies for prevention, diagnosis, and treatment, up-to-date research data on this subject are imperative.
